# Extended Spectrum Beta‐Lactamase Producing *Escherichia coli* in Pet Cats and Dogs in Central Peninsular Malaysia

**DOI:** 10.1002/vms3.70452

**Published:** 2025-06-13

**Authors:** Khaleeda Azalea Dzulkifli, Latiffah Hassan, Zunita Zakaria, Puteri Azaziah Megat Abdul Rani, Nur Indah Ahmad

**Affiliations:** ^1^ Department of Veterinary Pathology and Microbiology, Faculty of Veterinary Medicine Universiti Putra Malaysia Selangor Malaysia; ^2^ Department of Veterinary Laboratory Diagnostics, Faculty of Veterinary Medicine Universiti Putra Malaysia Selangor Malaysia; ^3^ Institute of Bioscience Universiti Putra Malaysia Selangor Malaysia; ^4^ Jade Hills Veterinary Hospital, Jalan Jade Hills Selangor Malaysia

**Keywords:** antimicrobial drug resistance, beta‐lactamases, cat, dog, *Escherichia coli*

## Abstract

**Background:**

Epidemiological study of pets in Malaysia as reservoirs for antimicrobial resistant bacteria is unknown.

**Objectives:**

This study aims to determine the extended‐spectrum beta‐lactamase (ESBL) producing *Escherichia coli* from rectal swabs pet cats and dogs in the central region of Peninsular Malaysia.

**Materials and Methods:**

A cross‐sectional study on physically healthy pet cats and dogs was conducted in the Klang Valley between 2019 and 2020. Rectal swabs were collected for ESBL‐producing *E*. *coli* identification and detection. A questionnaire was used to collect pet data, and univariable and multivariable analysis was conducted to determine risk factors ESBL‐producing *E. coli* detection.

**Results:**

A total of 160 rectal swabs were collected from physically healthy pets in the Klang Valley, with 6.8% (*n* = 11) dogs and 3.8% (*n* = 6) cats were positive for ESBL‐producing *E. coli* (10.6%). The highest resistance demonstrated by the ESBL isolates was towards ampicillin, cephalexin and cefotaxime (100%). Multiple‐drug resistance of the ESBL‐isolates was high, with 91.7% (*n* = 22) and 68.8% (*n* = 11) in dogs and cats. The most predominant ESBL genes was *bla*_CTX‐M_ (43.2%). None of these isolates carried *bla*_SHV_. Dogs were four times more likely to carry ESBL‐producing *E. coli* in their faeces compared to cats. ESBL‐producing *E. coli* carriage was positively associated in dogs, among pets sharing feeding area or pets with a history of gastrointestinal symptoms.

**Conclusion:**

ESBL‐producing *E. coli* in cats and dogs in Malaysia was determined at 10.6%, with dogs at a greater risk of carrying the bacteria. Co‐resistance to more than three types of tested antibiotics for the ESBL‐producing *E. coli* isolated was high. Pets act as reservoirs for antimicrobial resistance (AMR) and resistance genes that can be shared with human through interactions. Antimicrobial stewardship remains critical to safeguard not only antibiotics but also the health of pets and humans.

## Graphical abstract

• The prevalence of ESBL‐*E. coli* in cats and dogs in central Peninsular Malaysia was 10.6%.

• Dogs are at a greater risk of carrying the bacteria, and among pets sharing feeding area and pets with a history of gastrointestinal symptoms.

• Co‐resistance to more than three types of tested antibiotics for the ESBL‐*E. coli* isolated was high.

• The most predominant ESBL gene was *bla*_CTX‐M_.

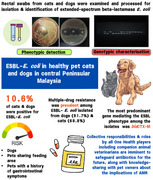

## 1 Introduction

Antimicrobial resistance (AMR) continues to be one of the major public health concerns worldwide. Studies on AMR across different landscapes of life encompassing samples originating from humans, animals, including wild animals, and the environment, particularly irrigation and streams, indicate the complexity of the problem. Unlike other diseases with visible signs and symptoms, patients infected with any multi‐drug resistant organisms oftentimes do not show any pathognomonic symptoms and can only be diagnosed upon failure of multiple treatments (Rahman et al. 2020).

The surge in the number of veterinary clinics in the central urban agglomeration of Peninsular Malaysia known as Klang Valley reflects a positive development in providing better veterinary services to the pets in the area (Malaysia Small Animal Veterinary Association n.d.) This allows easiest access for pet owners to obtain immediate veterinary treatment, encourages responsible pet ownership and improves pet welfare. The ease of access of pets to veterinary care could hypothetically, at the same time, increase the likelihood of antimicrobial drug consumption. This justifies the need to monitor the current situation and spread of AMR in companion animals in Malaysia. It will also help to conserve the efficacy of vital antimicrobial drugs for use in companion animals through a strengthened antimicrobial stewardship amongst small animal veterinarians.

The Malaysian Action Plan for AMR (2017–2021 and 2022–2026) includes a collaborative national AMR surveillance working group that incorporates the animal and environmental health sector, with focus on food‐producing animals and foods of animal origin (Ministry of Health Malaysia 2021). Surveillance of AMR in pets is not included in the national AMR monitoring programme. This may be due to prioritisation of costs and resources, leading to the lack of data on AMR from pets. The exclusion of pets from annual AMR surveillance programmes may risk underestimating the burden of AMR in pets, which could then lead to the spread and maintenance of AMR genes from a One Health perspective.

Compared to foodborne animals, pets are more often in close contact with their owners (Van Den Bunt et al. 2020), including those who are at risk of succumbing to non‐responding bacterial infections, such as immunosuppressed and elderly individuals. AMR in pathogenic or commensal bacteria isolated from pets has recently been included as part of the data deposited into centralised AMR surveillance databases in European countries (Nielsen et al. 2021)

Extended‐spectrum beta‐lactamase (ESBL) producing *Escherichia coli* is one of the indicator bacteria used in surveillance programmes worldwide to monitor the presence and spread of AMR (World Health Organization et al. 2019). Production of enzyme is one of the antimicrobial drug resistant mechanisms in bacteria which are able to lyse, inactivate or alter the target site in the bacterial cell, besides the alteration of the outer membrane permeability, alteration of target and efflux pump. ESBL is one of the enzymes responsible in antimicrobial drug resistant mechanism that enable the bacteria to withstand penicillin and monobactams, resulting in resistance towards third‐generation cephalosporins (Drawz and Bonomo 2010). Determination of ESBL‐producing *E. coli* and their susceptibility pattern will provide data on the emergence, spread and risk factors for multiple drug‐resistant bacteria at the human‐pets interface in Klang Valley.

## 2 Materials and Methods

### 2.1 Study Design

This was a cross‐sectional study to estimate the occurrence of ESBL‐producing *E*. *coli* in pet cats and dogs in the Klang Valley, located in the central of the West Coast of Peninsular Malaysia, consisting of main major cities with rapid of urbanisation (Abdul Rashid, 2017). (Veterinary clinics included in the study were based on convenience sampling method, following contact and consent approval by the owners. A total of 15 veterinary clinics agreed to participate as sampling premise, with 103 cats and 57 dogs were recruited between February 2019 and January 2020. The cats and dogs recruited were those that appeared healthy and not in any distressful conditions (critical/morbid/emergency case), to avoid discomfort or endangering the lives of the pets.

### 2.2 Phenotypic Identification of ESBL‐*E. coli*

#### 2.2.1 *E. coli* Isolation and Identification

Transport medium and sterile collection swabs were provided to the participating veterinary clinics. Rectal swabs were placed in Cary‐Blair transport media (Labchem Sdn. Bhd., Malaysia) and stored at 4°C before further processing. The swabs were incubated in buffered peptone water (Oxoid, UK) with 1 mg/L cefotaxime for 18 h at 37°C. The enriched samples were then streaked onto MacConkey (Oxoid, UK) agar supplemented with 1 mg/L of cefotaxime for another 18 h at 37°C. Three bacterial colonies growing on the selective MacConkey agar plates were identified as presumed ESBL‐producing bacteria and subjected to a series of biochemical tests (oxidase, indole, triple sugar iron, urea, SIM and citrate), following the standard by the Microbiology and Diagnostic Laboratory, Veterinary Medical Teaching Hospital, University of California, Davis for bacterial species identification. Presumptive ESBL‐producing bacterial identified *E. coli* were stored for further procedures.

#### 2.2.2 Confirmatory Test of ESBL Production by Double Disk Synergy Test (DDST)

Presumptive ESBL‐producing *E. coli* isolates were streaked on Mueller–Hinton (Oxoid, UK) agar plates at 1.5 × 10^8^ cfu/mL (0.5 McFarland Standard). Cefotaxime (30 µg) (Oxoid, UK) disk and ceftazidime (30 µg) (Oxoid, UK) disk were dispensed at 25 mm apart from the amoxicillin‐clavulanic acid disk (30 µg) (Oxoid, UK). The plates were incubated for 18 h at 37°C. Zone of inhibition (ZOI) was interpreted according to VET01‐S2 CLSI standard. Formation of a keyhole shape on the plate with cefotaxime‐amoxicillin‐clavulanic acid‐ceftazidime disc was interpreted as phenotypic confirmation of ESBL production by the bacterial lawn culture (Drieux et al. 2008; Rawat and Nair 2010).

#### 2.2.3 Quality Control

*E. coli* ATCC 25922 and *Klebsiella pneumoniae* ATCC 700603 served as quality control strains.

### 2.3 Antibiotic Susceptibility Testing

Susceptibility of the ESBL‐producing *E. coli* isolates from pets towards select antibiotics were determined by disk diffusion method (VET01‐S2, CLSI). The list of antibiotics tested were amoxicillin‐clavulanic acid (30 µg), tetracycline (30 µg), imipenem (10 µg), enrofloxacin (5 µg), cephalexin (30 µg), ampicillin (10 µg), nalidixic acid (30 µg), gentamicin (30 µg), ceftazidime (30 µg) and aztreonam (30 µg) (Oxoid, UK).

### 2.4 Multiple Antibiotic Resistance (MAR) Index Calculation

The MAR index is determined by using formula of *a/b* where ‘*a*’ is represented by the number of antibiotics that an isolate was resistant to; meanwhile, ‘*b*’ is the total number of antibiotics tested in the study (Buranasinsup et al. 2023; Mir et al. 2022).

### 2.5 ESBL Genes Identification

#### 2.5.1 Bacterial DNA Extraction and Amplification

Boiling method was conducted on isolates phenotypically identified as ESBL‐producing *E. coli*. A loopful of ESBL‐producing *E. coli* culture were suspended in 200 µL sterile distilled water and heated at 96°C for 10 min, cooled to room temperature for 10 min and centrifuged at 13,000 × *g* for 10 min. The supernatant was served as DNA template for PCR.

Multiplex PCR were performed to detect *bla*_TEM_, *bla*_CTX‐M_ and ESBL genes using primers as previously described (Aliyu et al. 2016; Kamaruzzaman et al. 2020) (Table 1). A PCR reaction cocktail was prepared with a total volume of 50 µL for each reaction, consisting of 25 µL MyTaq (Bioline, UK), 14 µL sterile distilled water, 1 µL of 20 µM primers (Apical Scientific Sdn. Bhd., Malaysia) and 5 µL bacterial DNA, with cycling conditions as described (Kamaruzzaman et al. (2020). *bla*_SHV_ was detected using uniplex PCR with 25 µL MyTaq (Bioline, UK), 18 µL sterile distilled water, 1 µL (20 µM *bla*_SHV_) primer and 5 µL bacterial DNA. One cycle of initial denaturation was conducted at 95°C for 1 min, followed by 30 cycles of denaturation at 95°C for 15 s, annealing at 60°C at 30 s, extension at 72°C for 10 s and a cycle of final extension at 72°C for 5 min.

**TABLE 1:** List of primers used in PCR to detect and confirm extended‐spectrum beta‐lactamase (ESBL) genes in ESBL producing *Escherichia coli* isolates from pet cats and dogs.

ESBL targeted gene	Primer	Sequence (5′–3′)	Size of product (bp)	GenBank accession no.
*bla*_TEM_	Forward	TCCTTGAGAGTTTTCGCCCC	643	EU352903
	Reverse	TGACTCCCCGTCGTGTAGAT		
*bla*_CTX‐M_	Forward	AAGCACGTCAATGGGACGAT	402	JN411912
	Reverse	GTTGGTGGTGCCATAGCCA		
*bla*_SHV_	Forward	CAATCACGACGGCGGAATCT	168	AB731686
	Reverse	GTGGGTCATGTCGGTACCAT		

The amplicons were separated by gel electrophoresis on 1.5% agarose gels in 5 µL gel stain (Yeastern Biotech, Taiwan) for 40 min at 100 V. The presence of bands on the gel were determined by AlphaImager Gel Imaging System (Bio‐Rad, USA).

#### 2.5.2 ESBL Genes Sequencing

The amplicons were for Sanger sequencing (Apical Scientific Sdn. Bhd., Malaysia). The set of primers used for PCR were used as templates, and the results were evaluated on the basis of GenBank (https://blast.ncbi.nlm.nih.gov/Blast.cgi). Three ESBL‐producing *E. coli* field isolates expressing ESBL genes (*bla*_TEM_, *bla*_CTX‐M_ and co‐detection *bla*_TEM_/*bla*_CTX‐M_) that have 90%–100% per identical strain in the GenBank (Table S1) were selected as positive controls, whereas distilled water was used as a negative control.

### 2.6 Study Instrument

A questionnaire with close‐ended questions was developed to collect information for analysing potential risk factors from the pet owners and divided into three sections: pet characteristics, pet lifestyle, including feeding and roaming behaviour, and hygiene practices. The questionnaire was translated into Malay, English and Mandarin languages to facilitate comprehension and participation of the pet owners, aligned to the main demographic backgrounds of people living in the Klang Valley. The questions were reviewed by experts consisting of one companion animal veterinarian, microbiologists and epidemiologists from the research team and pre‐tested to another set of pet owners to ensure face validity of the instrument.

### 2.7 Data Analysis

Data were organised in Microsoft Excel, and descriptive analysis were conducted. The variables were categorised according to species (canine or feline), age (young or adult), sex (male or female), score vaccination, deworming and neutering status (yes or no), raw‐based diet (yes or no), source of drinking water (tap or non‐tap water), milk consumption (yes or no), feeding area (indoor or outdoor), access of other animals in the feeding area (yes or no), presence of other animals in the house (yes or no), interaction between the sampled pet and other pets in the house (yes or no), hunting and roaming (yes or no), presence of litter box (yes or no), history of antibiotics uses (yes or no) and history of urinary tract infection (UTI) or GI symptoms (yes or no). Univariable logistic regression and multivariable logistic regression analysis were conducted to identify risk factors for ESBL‐producing *E. coli* carriage in the faeces of pet dogs and cats using Statistical Package for the Social Science (SPSS 25.0, IBM, NY, USA). A chi square test was conducted for univariable logistic regression analysis. Variables with a significance value at *p* ≤ 0.25 in univariable logistic regression analysis were included in multivariable logistic regression analysis using binary logistic model. The multivariable analysis was conducted using the backward Wald method and forward stepwise likehood ratio. The predictors were determined at *p* ≤ 0.05 and association strength was represented as odds ratio (OR). To fit the best model, Hosmer–Lemeshow test was applied. Haldane principle was used when one of the cells had zero value (Neuenschwander et al. 2000).

## 3 Results

A total of 160 owned cats (*n* = 103) and dogs (*n* = 57) were sampled throughout this study. Of all pets sampled, 10.6% (*n* = 17) were found to be positive for ESBL‐producing *E. coli*. Isolation of ESBL‐producing *E. coli* was more frequently detected in dogs (6.8%, *n* = 11) than cats (3.8%, *n* = 6). Pets identified positive for ESBL‐producing *E. coli* were in the age of 2 months old (*n* = 3), 1 year old (*n* = 4), 2 years old (*n* = 1), 3 years old (*n* = 2), 5 years old (*n* = 1), 6 years old (*n* = 1), 7 years old (*n* = 1), 9 years old (*n* = 1) and three other animals with no age data, and most were vaccinated (*n* = 10) and dewormed (*n* = 12). The reasons for visiting the veterinary clinic among pets with ESBL *E. coli* were either for vaccination, deworming or neuter appointments seeking veterinary consultation and for pet boarding. None of these were statistically associated with positive isolation of ESBL‐producing *E. coli* from the rectal swabs.

Of the 17 ESBL‐producing *E. coli* positive pets, 12 had access to outdoors (8 dogs, 4 cats), whereas 5 (4 dogs, 1 cat) were kept strictly indoors. A total of 11 owners reported previous exposure of their pets to antibiotic treatment, where 9 (5 dogs, 4 cats) of the pets had received antibiotics in the past 1–3 (5 dogs, 4 cats) months before sampling, whereas 1 pet dog was a year before, and another pet cat owner was unable to recall the exact timeline. No data on the specific antibiotics name of the drug, clinical symptoms and duration of the treatment were recorded, as the questionnaire were designed to sought for a general context of antibiotic exposure (Table S2).

Univariable analysis indicated variables associated with positive isolation of faecal ESBL‐producing *E. coli* from the pets in this study (Tables 2 and 3). Dogs with no gastrointestinal symptoms were identified to have higher odds of having ESBL‐producing *E. coli* isolated from the rectal swabs. Meanwhile, cats that were fed outdoor and with no access to litter box were associated with detection of ESBL‐producing *E. coli* in cats. Sex and neuter status of the animals were similarly distributed regardless of ESBL‐producing *E. coli* detection status. Raw feed consumption was not found to be associated with isolation of ESBL‐producing *E. coli* among the pets in the current study. The age of pets when compared between the young and adult age group for cats and dogs showed no significant statistical difference regarding ESBL‐producing *E. coli* detection from both age groups, despite the frequency were higher in cats less than one year of age when compared to other years of age recorded.

**TABLE 2:** Univariable and multivariable logistic regression analysis to determine variables associated with carriage of extended‐spectrum beta‐lactamase (ESBL)‐producing *Escherichia coli* among pet dogs visiting veterinary clinics in central Peninsular Malaysia.

Variable	Frequency (*n*)	ESBL‐*E. coli* isolation (%)	Univariable logistic regression analysis OR (95% CI)	*p* Value
**Pet age**				
Adult (>7 months)	48	9 (18.75)	1.23 (0.15–6.27)	0.79
Young (<7 months)	9	2 (22.22)	1.00^a^	
**Sex**				
Male	21	4 (19.05)	0.97 (0.22–3.88)	0.99
Female	36	7 (19.44)	1.00^a^	
**Vaccination status**				
Yes	46	7 (15.2)	0.32 (0.71–1.54)	0.15
No	11	4 (36.3)	1.00^a^	
**Neutering status**				
Yes	23	3 (13.0)	0.49 (0.09–2.06)	0.35
No	34	8 (23.5)	1.00^a^	
**Deworming status**				
Yes	46	7 (15.2)	0.32 (0.71–1.53)	0.15
No	11	4 (36.3)	1.00^a^	
**Type of diet**				
Raw diet	2	0 (0)	4.09 (0.09–2.06)	0.40
Non‐raw diet	55	11 (20.0)	1.00^a^	
**Drinking water source**				
Tap	18	5 (27.7)	2.08 (0.50–8.42)	0.30
Other than tap water	39	6 (15.3)	1.00^a^	
**Feed milk to pet**				
Yes	8	2 (25.0)	1.47 (0.17–8.34)	0.66
No	49	9 (18.3)	1.00^a^	
**Feeding area**				
Indoor	38	7 (18.4)	0.84 (0.21–3.77)	0.81
Outdoor	19	4 (21.9)	1.00^a^	
**Access other animals at feeding area**				
Yes	16	2 (12.5)	0.51 (0.06–2.57)	0.46
No	41	9 (21.9)	1.00^a^	
**Presence of other animals in the household**				
Yes	32	7 (21.88)	1.46 (0.37–6.40)	0.60
No	25	4 (16.0)	1.00^a^	
**Pet contacts with other animals**				
Frequent	16	2 (12.5	0.51 (0.06–2.51)	0.46
Non‐frequent	41	9 (21.9)	1.00^a^	
**Pest hunting behaviour**				
Yes	33	6 (18.1)	0.84 (0.21–3.42)	0.80
No	24	5 (29.8)	1.00^a^	
**Pet roaming outdoors**				
Yes	23	4 (17.3)	0.81 (0.18–3.22)	0.79
No	34	7 (20.5)	1.00^a^	
**Litter box provided**				
Yes	30	6 (20.0)	1.09 (0.28–4.42)	0.90
No	27	5 (18.5)	1.00^a^	
**Owner report of antibiotics use for pets**				
Yes	21	3 (14.2)	0.58 (0.11–2.47)	0.50
Never	36	8 (22.2)	1.00^a^	
**Owner report of UTI symptoms in pets**				
Yes	3	0 (0)	2.04 (0.64–29.03)	0.60
No	54	17 (20.3)	1.00^a^	
**Owner report of gastrointestinal symptoms in pets**				
No	36	10 (27.7)	7.48 (1.116–176.2)^*^	**0.004** ^*^
Yes	21	1 (4.7)	1.00^a^	

Abbreviations: OR, odds ratio; UTI, urinary tract infection.

^a^ Reference variable.

* Significance level at *p* ≤ 0.05.

**TABLE 3:** Univariable and multivariable logistic regression analysis to determine variables associated with carriage of extended‐spectrum beta‐lactamase (ESBL) producing *Escherichia coli* among pet cats visiting veterinary clinics in central Peninsular Malaysia.

Variable	Frequency (*n*)	ESBL‐*E. coli* isolation (%)	Univariable logistic regression analysis OR^b^ (95% CI)	*p* Value
**Pet age**				
Adult (>7 months)	10	1 (10.0)	1.94 (0.07–16.11)	0.57
Young (<7 months)	93	5 (5.3)	1.00^a^	
**Sex**				
Male	50	5 (10.0)	5.69 (0.75–139.9)	0.10
Female	53	1 (1.8)	1.00^a^	
**Vaccination status**				
Yes	66	3 (4.5)	0.54 (0.08–3.31)	0.49
No	37	3 (8.1)	1.00^a^	
**Neutering status**				
Yes	50	3 (6.0)	1.66 (0.17–6.46)	0.94
No	53	3 (5.6)	1.00^a^	
**Deworming status**				
Yes	79	5 (6.3)	1.54 (0.20–38.5)	0.77
No	24	1 (4.1)	1.00^a^	
**Type of diet**				
Raw diet	10	2 (20.0)	5.40 (0.61–35.61)	0.12
Non‐raw diet	93	4 (4.3)	1.00^a^	
**Drinking water source**				
Tap	49	4 (8.1)	2.29 (0.38–18.16)	0.38
Other than tap water	54	2 (3.7)	1.00^a^	
**Feed milk to pet**				
Yes	28	3 (10.7)	2.84 (0.46–17.53)	0.25
No	75	3 (4.0)	1.00^a^	
**Feeding area**				
Outdoor	23	5 (21.7)	21.06 (2.722–527.4)	**0.02** ^*^
Indoor	80	1 (1.2)	1.00^a^	
**Access other animals at feeding area**				
Yes	54	4 (7.4)	1.86 (0.31–15.17)	0.51
No	49	2 (4.0)	1.00^a^	
**Presence of other animals in the household**				
Yes	56	5 (8.9)	4.27 (0.56–105.1)	0.19
No	47	1 (2.1)	1.00^a^	
**Pet contacts with other animals**				
Frequent	41	3 (7.3)	1.54 (0.25–9.41)	0.62
Non‐frequent	62	3 (4.8)	1.00^a^	
**Pest hunting behaviour**				
Yes	84	5 (5.9)	1.13 (0.14–28.54)	0.98
No	19	1 (5.2)	1.00^a^	
**Pet roaming outdoors**				
Yes	51	1 (1.9)	0.19 (0.007–1.43)	0.12
No	52	5 (9.6)	1.00^a^	
**Litter box provided**				
No	24	5 (20.8)	19.74 (2.558–493.4)	**0.002** ^*^
Yes	79	1 (1.2)	1.00^a^	
**Owner report of antibiotics use for pets**				
Yes	47	5 (10.6)	6.44 (0.85–158.4)	0.007
Never	56	1 (1.7)	1.00^a^	
**Owner report of UTI symptoms in pets**				
Yes	8	0 (0)	2.29 (0.08–19.32)	0.50
No	95	6 (6.3)	1.00^a^	
**Owner report of gastrointestinal symptoms in pets**				
Yes	33	2 (6.0)	1.06 (0.13–6.31)	0.92
No	70	4 (5.71)	1.00^a^	

Abbreviations: OR, odds ratio; UTI, urinary tract infection.

^a^ Reference variable.

* Significance level at *p* ≤ 0.05.

Multivariable logistic regression with the best‐fit model indicated variable associated with higher odds of isolation ESBL‐producing *E. coli* from dogs is not having gastrointestinal symptoms before (*p* = 0.03, OR: 11.82, 95% CI: 1.24–112.75) and outdoor feeding area (*p* = 0.003, OR: 53.10, 95% CI: 3.75–752.05) associated with ESBL‐producing *E. coli* detection in cats. Dog species (*p* = 0.02, OR: 4.73, 95% CI: 1.25–17.86), presence of other animals at the feeding area (*p* = 0.04, OR: 5.49, 95% CI: 1.02–29.74) and not having GI symptoms before (*p* = 0.01. OR: 7.42, 95% CI: 1.39–39.50) were the risk factors associated with ESBL‐producing *E. coli* in both species (Table S8). The complete characteristics of pets and responses of their owners to the questionnaire are summarised in Table S3, S4 and S5.

**FIGURE 1:**
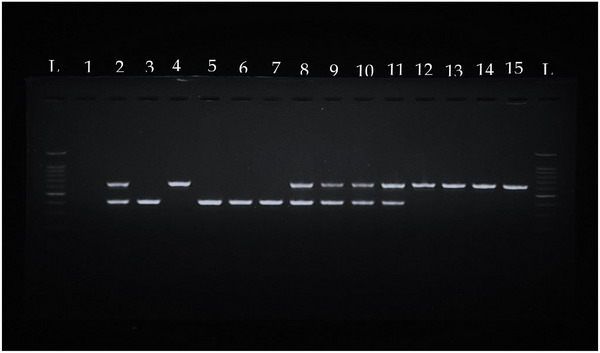
Detection of ESBL genes in phenotypically confirmed ESBL‐producing *Escherichia coli* from cats and dogs. Lane L, ladder (100 bp); Lane 1, sterile distilled water; Lane 2–4, positive controls (*bla*_TEM_/_CTX‐M_), (*bla*_CTX‐M_), (*bla*_TEM_); Lane 5–7, *bla*_CTX‐M_ positive isolates; Lane 8–11, *bla*_TEM_/_CTX‐M_ isolates; and Lane 12–15, *bla*_TEM_ isolates.

A total of 40 ESBL‐producing *E. coli* isolates were obtained from 11 dogs and 6 cats as 2 or 3 single colonies were collected in each sample. Three types of ESBL genes, *bla*_TEM_, *bla*_CTX‐M_ and *bla*_TEM/CTX‐M_, were detected from 37 ESBL‐producing *E*. *coli* isolated (Figure 1), Table S6 and S7. None of the ESBL‐producing *E. coli* isolates carried *bla*_SHV_ genes. *bla*_TEM_ was the most predominant ESBL gene in dogs (*n* = 11/24 isolates) and *bla*_CTX‐M_ (*n* = 8/13 isolates) in cats. Three isolates initially phenotypically confirmed as ESBL‐producing *E. coli*, which originated from the rectal swab of a cat, lack all ESBL genes investigated in this study.

There were 21 different combinations of co‐resistance characterised from the ESBL‐producing *E*. coli isolates, with 33 multiple‐drug resistant (MDR) isolates. MDR in this study is defined by isolates that resistant to more than three antimicrobial categories. Of these, 68.8% (*n* = 11/16) and 91.6% (*n* = 22/24) were isolates from cats and dogs, respectively. The most common co‐resistance carried by ESBL *E. coli* from dogs were CL‐AMP‐TE‐CN‐CTX‐ATM, whereas in cats were CL‐AMC‐CTX (Table 4, Table S8). All ESBL‐*E. coli* isolates remained susceptible to imipenem. The MAR index across all isolates were between 0.36 and 0.90, with the most frequent MAR index in dogs being 0.72 (7 isolates). In cats, the most frequent MAR index were 3 isolates each for 0.45, 0.54 and 0.63, and 1 isolate had an MAR index of 0.72 (Table 5).

**TABLE 4:** Multiple‐drug resistance profile observed in extended‐spectrum beta‐lactamase (ESBL)‐producing *Escherichia coli* isolated from cats and dogs in the Klang Valley.

No.	Antibiotic resistance profile
1.	Aminoglycoside‐cephalosporins‐monobactam‐penicillin‐tetracycline
2.	Aminoglycoside‐cephalosporins‐monobactam‐penicillin‐quinolone‐tetracycline
3.	Aminoglycoside‐cephalosporins‐monobactam‐penicillin‐quinolone‐tetracycline
4.	Aminoglycoside‐cephalosporins‐penicillin‐tetracycline
5.	Cephalosporins‐monobactam‐penicillin‐quinolone‐tetracycline
6.	Cephalosporins‐monobactam‐penicillin‐tetracycline
7.	Cephalosporins‐monobactam‐penicillin‐quinolone
8.	Cephalosporins‐penicillin‐tetracycline

**TABLE 5:** The multiple antibiotic resistance (MAR) index of extended‐spectrum beta‐lactamase (ESBL)‐ *Escherichia coli* in pet cats and dogs.

Dog		Cat	
MAR index = *a/b*	Number of isolates	MAR index = *a/b*	Number of isolates
0.45	1	0.36	2
0.54	5	0.45	3
0.63	2	0.54	3
0.72	7	0.63	3
0.81	5	0.72	1
0.90	2		

*Note*: *b* is the total number of antibiotics used (*n* = 11).

## 4 Discussion

Research on AMR to incorporate pets have increasingly become an emerging theme across the globe for the past few years (Marco‐fuertes et al. 2022; Salgado‐caxito et al. 2021). Despite not usually included in routine national AMR surveillance programmes, recent reports on difficult‐to‐treat common bacterial infections in pets should raise the alarm on the importance of incorporating pets into national AMR monitoring programmes (Lagana et al. 2023). Previous reports from other countries concluded pets as plausible carriers for antimicrobial‐resistant bacteria with potential impact on the epidemiology of AMR between humans and animals due to frequent and close interactions of pets with their owners sharing the same living environment (Guardabassi et al. 2004; Pérez‐Serrano et al. 2020).

In this study, approximately 1 in 10 of owned cats and dogs sampled from veterinary clinics in the central region of Peninsular Malaysia harboured ESBL‐producing *E. coli*. Compared to reports from the United States (Seo 2023) and dogs in Thailand (25.84%, *n* = 23/89) (Thepmanee et al. 2018), the reported prevalence in the current study is low and comparable to the findings in another study in Guadeloupe, Caribbeans (Gruel et al. 2022). However, the differences would mostly be attributed by the employment of different diagnostic methods to detect ESBL‐producing bacteria and incorporation of other Enterobacterales, including *K. pneumoniae* (Seo 2023; Thepmanee et al. 2018).

The neighbouring country, Indonesia, reported 9.41% (*n* = 8/85) isolation rate of ESBL‐producing *E. coli* in companion dogs (Kristianingtyas et al. 2021), meanwhile 5.63% (*n* = 4/71) in cats (Farizqi et al. 2023). In Thailand, the isolation rate of ESBL‐producing *E. coli* in cats and dogs is slightly higher (43.06%, *n* = 31/72 isolates) (Nittayasut et al. 2021) than current study (38.5%, *n* = 40/104). Determination of ESBL‐producing *E. coli* between this study and neighbouring countries was similar, which may be facilitated by the similar veterinary practices and regulations between these countries. However, there is limited data available for detection of ESBL‐producing *E. coli* in pet cats and dogs from other countries in Southeast Asia.

ESBL‐producing *E. coli* was predominantly isolated from dogs compared to cats, despite the higher number of cats sampled. This was also observed in previous studies (Hordijk et al. 2013; Melo et al. 2018; Sfaciotte et al. 2021; Van Den Bunt et al. 2020). Most of the dogs in the current study that carried ESBL‐producing *E. coli* were semi‐indoor pets. The exposure of dogs to ESBL‐producing *E. coli* in the environment mostly were suggested to be associated with their routine dog walking activity (Mccune and Promislow 2021). Dogs can come into contact with various microorganisms, including resistant bacteria, along their walking route with water and soil contaminated with animal faeces containing resistant bacteria (Garfield and Walker 2008; Ortega‐Paredes et al. 2019) as well as contact with other dogs (Westgarth et al. 2007). Dogs and potentially other pets with access to outdoors could further disseminate resistant bacteria from their household to the environment and vice versa during walking (Leite‐Martins et al. 2015).

Having a communal feeding area for pets in the household was associated with the occurrence of ESBL‐producing *E. coli* in the pet's faeces. ESBL bacteria can be transmitted through saliva (Founou et al. 2016; Riwu et al. 2020) while sharing the feeding or water bowls as well as through the pet food served. The transmission of ESBL bacteria was shown to be transmitted between domestic pets and livestock animals with close contact living under the same, shared same environment (De Oliveira et al. 2016).

Placing feeding bowls outdoors could attract pests such as rodents to frequent the communal area where the pets could come into contact with the rats or the rats’ excrements. This may be considered as another pathway for AMR spread in pets, as rats were reported to harbour ESBL‐producing *E. coli* (Ho et al. 2015; Onanga et al. 2020). From the One Health perspective, this is especially important when the human‐pet interactions involve those with weak immune system, including infants, the elderly and immunosuppressed individuals (Damborg et al. 2016).

No history of gastrointestinal symptoms experienced by dogs, as reported by the owners in this study, were associated to the isolation of ESBL‐producing *E. coli*. A serious risk from the animals that appeared to be healthy, which they may have the potential in disseminating ESBL bacteria, whereas being physically fine. In addition, this may pose another health risk to those handling faeces of their pets to contract the bacteria if there is lack of hygiene practices, especially when the pets experience diarrhoea (Cui et al. 2022; Piccolo et al. 2020; Tudu et al. 2022; Zhou et al. 2022).

Pet cats and dogs with UTI were often reported to be infected with uropathogenic *E*. *coli* and ESBL‐producing *E. coli* (Aurich et al. 2022; Darwich et al. 2021; Huber et al. 2013; Kuan et al. 2016; Zogg et al. 2018). Detection of ESBL‐producing *E. coli* were also determined in healthy dogs in Turkey (Kaplan and Gulaydin 2023). Both patients with UTI nor animals that are not having any symptoms of UTI may disseminate ESBL bacteria to their surroundings; thus, good hygiene practice is important in handling pet animals by the owners. Findings from this study were unable to observe any association between UTI symptoms and isolation of ESBL‐producing *E. coli* from pets. This mostly could be as our study focused on healthy pets and the type of sample used was faeces instead of urine.

Shedding of ESBL bacteria can be associated with feeding pets with commercial and non‐commercial raw feed derived from poultry meat, pork, beef and fish (Baede et al. 2017; Ramos et al. 2022). This, as ESBL‐bacteria, was shown to contaminate raw meat‐based diet (Hellgren et al. 2019; Nüesch‐Inderbinen et al. 2019; van Bree et al. 2018) and meat by‐product for pet diets (Bacci et al. 2019). However, feeding pets with raw meat‐based diet was not associated with pets found to be positive with ESBL‐producing *E. coli* in this study. This is most likely as the practice of feeding raw feed to pets was found to be uncommon among pet owners in this study. This could either be a result of convenient sampling method employed or that it reflects the actual situation whereby raw pet feeding is not common among pet owners in central region of Malaysia, both of which requires other study to elucidate further.

The age of the cats and dogs studied did not influence the isolation of ESBL‐producing *E. coli* from their faeces. A retrospective study on diagnostic laboratory data from Indiana, United States, showed that dogs of 10 years and older were more likely to carry AMR *E. coli* than other age groups (Ekakoro et al. 2022). This was suggested to possibly due to prior use of antimicrobial drugs in previous years of life (Wedley et al. 2017) or geriatric factors which predispose the animals to infection and requires more use of antimicrobial drugs. The disparity in the findings could mostly be due to the smaller sample size in the present study and requires further investigation with the higher number of pets to be included.

The most prevalent ESBL gene in dogs was *bla*_TEM_, similar to the report by (Meireles et al. (2015) and sheltered dogs in Japan (Hata et al. 2022), whereas in cats was *bla*_CTX‐M_. *bla*_TEM_ is responsible for ampicillin resistance in Enterobacteriaceae (Heinz 2018) whereas *bla*_CTX‐M_ mediates resistance in cefotaxime and other third generation cephalosporins (Cavalieri et al. 2005). These findings, taken together with the report that *bla*_TEM_ was the most commonly identified ESBL gene of clinical *E*. *coli* isolates from hospital patients in Malaysia, warrants the urgency of ensuring implementation of antimicrobial stewardship in both animal and human health sectors (Thong et al. 2009). Further study to investigate any association of ESBL genes originating from pets and humans in close contact will provide better evidence to imply any link in the complex epidemiology of AMR from the local context.

Besides *bla*_TEM_, *bla*_CTX‐M_ was also commonly isolated in this study. It is comparable to the study by Ortega‐Paredes et al. (2019) and slightly higher than reported by Rumi et al. (2019) in Argentina. The ESBL genes studied were not detected in three isolates that were phenotypically typed as ESBL producers. These three isolates may harbour other less common variants of ESBL genes that were not tested in the study, which includes *bla*_PER_, *bla*_VEB_, *bla*_GES_, *bla*_BES_ and *bla*_TLA_ (Bajpai et al. 2017) previously only detected in humans (Naas et al. 2008; Poirel et al. 2008). Bacterial isolates losing their resistance genes along the laboratory procedures could also result in such observation as previously reported (Schwan et al. 2021).

Sharing of similar genotypic and phenotypic of ESBL characteristics between pet animals and their owners (Hong et al. 2020) also between pet animals and the environments (Timofte et al. 2016) showed the importance of pet animals as the bacterial reservoirs. Moreover, a study has determined a potential antibiotic resistance transferability of bacteria in poultry to human pathogenic bacteria through horizontal gene transfer (Malik et al. 2022). The antimicrobial resistance gene (ARG) that was included in this study, *bla*_TEM_, is one of the gene studied by Pérez‐Serrano et al. (2020), which their results showed the pet dogs and their owners shared ARG profiles similarities, suggesting that close interaction between human and animals may become possible route in shading ESBL bacteria.

The most common AMR pattern among ESBL bacteria isolates in this study was towards cephalexin, ampicillin, tetracycline, gentamicin, cefotaxime and aztreonam. This is similar with the findings of a study on bacterial isolates from diseased pet samples in Malaysia, with *E*. *coli* isolates to be most resistant towards doxycycline, tetracycline and cephalexin. MDR isolates from diseased cats were reported to be higher than dogs, in contrast to the findings in the current study, where MDR was more prevalent among isolates from healthy dogs (Haulisah et al. 2022).

The predominant MAR index in the dog isolate is 0.72, higher than that reported by (Matos et al. 2023). Meanwhile, the MAR index in cats was comparable with that reported in ESBL‐*E. coli* collected from the oral cavity of domestic cats in Brazil (Souza Andrade et al. 2019). Bacterial isolates with MAR index > 0.2 are associated with faecal contamination sources or previous use or exposure to antibiotics (Afunwa et al. 2020; Khan et al. 2015). Judicious use of antibiotics need to be emphasised among veterinary practitioners, and effective knowledge sharing on the appropriate use of antibiotics to the pet owners must be delivered by the to prevent the misuse and overuse of antibiotics in pets.

Ampicillin is commonly prescribed by the veterinarian as first line drugs during treatment; however, for ESBL bacteria, it is ineffective (Spohr et al. 2012). This further limits the option of treatment available for pets, which also may foster the use of stronger groups of antibiotics. Justified use of antibiotics permitted for therapeutic use in pets in Malaysia can be based on the Malaysian Veterinary Antimicrobial Guidelines (MVAG). Imipenem was the only antibiotic that remains effective to all of the ESBL‐*E. coli* isolates in this study. It is classified as one of the last resort antibiotics, with use limited for critically ill human patients. It is not listed to be used in animals according to the MVAG, in an effort to encourage good antimicrobial stewardship programme among the veterinarians (Malaysian Veterinary Antimicrobial Guidelines (MYVAG) 2021). This is considered vital in preserving critically important antimicrobials from a One Health perspective, which requires continuous and strong commitment from both human and animal health sectors.

## 5 Limitations

This study captured a snapshot of AMR represented by ESBL‐producing *E. coli* in healthy, owned cats and dogs in an urbanised area in central Peninsular Malaysia with high number of veterinary clinics per area. Although this may suggest that the pets in the area have better access and exposure to antibiotics from antimicrobial prescription, the study is unable to elucidate this hypothesis further due to the nature of the sampling strategy and study design. Information bias that may arise because of the owners not being able to recall and report accurately on previous use of antibiotics may influence the conclusions from the results obtained.

## 6 Conclusion

This is the first report about the prevalence of ESBL‐producing *E. coli* in pet cats and dogs in Malaysia, with 10.6% of pet cats and dogs in the Klang Valley harbours the multiple drug resistant bacteria in their faeces. Considering the health and well‐being of pets, which will also require antibiotic for therapeutic purposes, continuous surveillance should incorporate pets, which will allow better representation of the AMR situation in pets, as well as ensuring there will be appropriate antibiotic treatment available for humans and animals.

## Author Contributions

**Khaleeda Azalea Dzulkifli**: investigation, writing – original draft, writing – review and editing, visualization, methodology, formal analysis, data curation, project administration. **Latiffah Hassan**: funding acquisition, validation, supervision, resources, writing – review and editing, conceptualization, methodology. **Zunita Zakaria**: funding acquisition, validation, writing – review and editing, supervision, resources, conceptualization, methodology. **Puteri Azaziah Megat Abdul Rani**: conceptualization, funding acquisition, writing – review and editing, resources, methodology. **Nur Indah Ahmad**: conceptualization, funding acquisition, investigation, writing – original draft; writing – review and editing, methodology, validation, project administration, supervision, data curation, resources.

## Ethic Statement

The authors confirm that the ethical policies of the journal, as noted on the journal's author guidelines page, have been adhered to and the appropriate ethical review committee approval has been received (UPM/IACUC/AUP‐R095/2018). The Institutional Animal Care and Use Committee (IACUC) Universiti Putra Malaysia guidelines for the Care and Use of Animals were followed.

## Conflicts of Interest

The authors declare no conflicts of interest.

## Peer Review

The peer review history for this article is available at https://www.webofscience.com/api/gateway/wos/peer‐review/10.1002/vms3.70452.

## Supporting information




**Table S1**: Reference strains for ESBL producing *Escherichia coli* isolates.
**Table S2**: Summary of questionnaire response as reported by owners.
**Table S3**: Multivariable logistic regression of risk factors associated of ESBL‐*E. coli* carriage in pet dogs.
**Table S4**: Multivariable logistic regression of risk factors associated of ESBL‐*E. coli* carriage in pet cats.
**Table S5**: Multivariable logistic regression of risk factors associated of ESBL‐*E. coli* carriage in sampled pet cats and dogs.
**Table S6**: ESBL genes identified in ESBL producing *E. coli* isolated from healthy pet cats and dogs.
**Table S7**: ESBL‐genes detected in individual ESBL producing *Escherichia coli* isolates from pet cats and dogs.
**Table S8**: Multiple‐drug resistance profile observed in ESBL producing *E. coli* isolated from cats and dogs in the Klang Valley.

## Data Availability

The data that support the findings of this study are available from the corresponding author upon reasonable request.
