# Supplementary material for: Extended Spectrum Beta‐Lactamase Producing Escherichia coli in Pet Cats and Dogs in Central Peninsular Malaysia
Source: Vet Med Sci. 2025 Jun 13;11(4):e70452. doi: 10.1002/vms3.70452 (PMC12163349; doi:10.1002/vms3.70452)
Supplement: Supplementary file 1 — Table S1: Reference strains for ESBL producing Escherichia coli isolates. Table S2: Summary of questionnaire response as reported by owners. Table S3: Multivariable logistic regression of risk factors associated of ESBL‐E. coli carriage in pet dogs. Table S4: Multivariable logistic regression of risk factors associated of ESBL‐E. coli carriage in pet cats. Table S5: Multivariable logistic regression of risk factors associated of ESBL‐E. coli carriage in sampled pet cats and dogs. Table S6: ESBL genes identified in ESBL producing E. coli isolated from healthy pet cats and dogs. Table S7: ESBL‐genes detected in individual ESBL producing Escherichia coli isolates from pet cats and dogs. Table S8: Multiple‐drug resistance profile observed in ESBL producing E. coli isolated from cats and dogs in the Klang Valley. [file VMS3-11-e70452-s001.docx]

**Supplementary data**

**STable 1.** Reference strains for ESBL producing *Escherichia coli* isolates.

| ESBL gene | Reference strain accession number |
| --- | --- |
| *bla*_TEM_ | - NG_062250.1 - MG860488.1 |
| *bla*_CTX-M_ | - MK034764.1 - KP309815.1 - OM326859.1 |
| *bla*_TEM/CTX-M_ | - MZ310399.1 (*bla*_CTX-M_) - MK034764.1 (*bla*_CTX-M_) - KP853092.1 (*bla*_TEM_) - MT387455.1 (*bla*_TEM_) |

| STable 2. Summary of questionnaire response as reported by owners | | | | | |
| --- | --- | --- | --- | --- | --- |
| Response | Dog (n=57) | | Cat (n=103) | | ESBL + |
|  | n (%) | ESBL+ | n (%) | ESBL+ | n (%) |
| Age  Young (< 7 months)  Adult (> 7 months) | 9 (15.8)  48 (84.2) | 2 (22.22)  9 (18.75) | 10 (9.7)  93 (90.3) | 1 (10.0)  5 (5.38) | 3 (17.65)  14 (82.35) |
| Sex  Male  Female | 21 (36.8)  36 (63.2) | 4 (19.05)  7 (19.44) | 50 (48.5)  53 (51.5) | 5 (10.0)  1 (1.89) | 9 (52.94)  8 (47.06) |
| Vaccination status  Yes  No | 46 (80.7)  11 (19.3) | 7 (15.22)  4 (36.36) | 66 (64.1)  37 (35.9) | 3 (4.55)  3 (8.11) | 10 (58.82)  7 (41.18) |
| Deworming status  Yes  No | 46 (80.7)  11 (19.3) | 7 (15.22)  4 (36.36) | 79 (76.7)  24 (23.3) | 5 (6.33)  1 (4.17) | 12 (70.59)  5 (29.41) |
| Neutering status  Yes  No | 23 (40.4)  34 (59.6) | 3 (13.04)  8 (23.53) | 50 (48.5)  53 (51.5) | 3 (6.00)  3 (5.66) | 6 (35.29)  11 (64.71) |
| Type of diet  Raw meat  Non-raw meat | 2 (3.5)  55 (96.5) | 0 (0)  11 (20.0) | 10 (9.7)  93 (90.3) | 2 (20.0)  4 (4.30) | 2 (11.76)  15 (88.24) |
| Feeding area  Indoor  Outdoor | 38 (66.7)  19 (33.3) | 7 (18.42)  4 (21.95) | 80 (77.7)  23 (22.3) | 1 (1.25)  5 (21.74) | 8 (47.06)  9 (52.94) |
| Other animal allowed at feeding area  Yes  No | 16 (28.1)  41 (71.9) | 2 (12.5)  9 (21.95) | 54 (52.4)  49 (47.6) | 4 (7.41)  2 (4.08) | 6 (35.29)  11 (64.71) |
| Drinking water source  Tap water  Non-tap water | 18 (31.6)  39 (68.4) | 5 (27.78)  6 (15.38) | 49 (47.6)  54 (52.4) | 4 (8.16)  2 (3.70) | 9 (52.94)  8 (47.06) |
| Feed milk to pet  Yes  No | 8 (14)  49 (86) | 2 (25.0)  9 (18.37) | 28 (27.2)  75 (72.8) | 3 (10.71)  3 (4.0) | 5 (29.41)  12 (70.59) |
| Presence of other animal  Yes  No | 32 (56.1)  25 (43.9) | 7 (21.88)  4 (16.0) | 56 (54.4)  47 (45.6) | 5 (8.93)  1 (2.13) | 12 (70.59)  5 (29.41) |
| Pet close contact with other pet in the house  Frequent  Non-frequent | 16 (28.1)  41 (71.9) | 2 (12.5)  9 (21.95) | 41 (39.8)  62 (60.2) | 3 (7.32)  3 (4.84) | 5 (29.41)  12 (70.59) |
| Pest hunting  Yes  No | 33 (57.9)  24 (42.1) | 6 (18.18)  5 (29.83) | 84 (81.6)  19 (18.4) | 5 (5.95)  1 (5.26) | 11 (64.71)  6 (35.29) |
| Pet roaming outdoors  Yes  No | 23 (40.4)  34 (59.6) | 4 (17.39)  7 (20.59) | 51 (49.5)  52 (50.5) | 1 (1.96)  5 (9.62) | 5 (29.41)  12 (70.59) |
| Litter box  Yes  No | 30 (52.6)  27 (47.4) | 6 (20.0)  5 (18.52) | 79 (76.7)  24 (23.3) | 1 (1.27)  5 (20.83) | 7 (41.18)  10 (58.82) |
| Exposure to antibiotics  Yes  Never | 21 (36.8)  36 (63.2) | 3 (14.29)  8 (22.22) | 47 (45.6)  56 (54.4) | 5 (10.64)  1 (1.79) | 8 (47.06)  9 (52.94) |
| Encounter UTI symptoms  Yes  No | 3 (5.3)  54 (94.7) | 0 (0)  11 (20.37) | 8 (7.8)  95 (92.2) | 0 (0)  6 (6.32) | 0 (0)  17 (100) |
| Encounter GI symptoms  Yes  No | 21 (36.8)  36 (63.2) | 1 (4.76)  10 (27.78) | 33 (32)  70 (68) | 2 (6.06)  4 (5.71) | 3 (17.65)  14 (82.35) |

**STable 3.** Multivariable logistic regression of risk factors associated of ESBL-*E. coli* carriage in pet dogs.

| Risk factor | (B) | S.E. | Wald | OR^a^ | CI (95%) | *p-*  value |
| --- | --- | --- | --- | --- | --- | --- |
| Presence of gastrointestinal associated symptoms (No) | 2.470 | 1.151 | 4.609 | 11.824 | 1.240-112.749 | 0.032* |
| Vaccination status (Yes) | -1.660 | 0.879 | 3.565 | 0.190 | 0.34-1.065 | 0.059 |
| Pet wanders (Yes) | 0.780 | 0.770 | 1.027 | 2.182 | 0.482-9.868 | 0.311 |

^a^ OR= odds ratio

*Significance level at *p*≤0.05.

**STable 4.** Multivariable logistic regression of risk factors associated of ESBL-*E. coli* carriage in pet cats.

| Risk factor | (B) | S.E. | Wald | OR^a^ | CI (95%) | *p-*  value |
| --- | --- | --- | --- | --- | --- | --- |
| Feeding area (Outdoor) | 3.972 | 1.352 | 8.628 | 53.10 | 3.750-752.054 | 0.003* |
| Food consumption  (Raw diet) | 32.473 | 1.537 | 0.000 | 1.223E+14 | 0.000 | 0.994 |
| Exposure to antibiotics (Yes) | 47.415 | 5469.371 | 0.000 | 3.909E+20 | 0.000 | 0.993 |
|  |  |  |  |  |  |  |

^a^ OR= odds ratio

*Significance level at *p*≤0.05.

**STable 5.** Multivariable logistic regression of risk factors associated of ESBL-*E. coli* carriage in sampled pet cats and dogs.

| Risk factor | (B) | S.E. | Wald | OR^a^ | CI (95%) | *p-*  value |
| --- | --- | --- | --- | --- | --- | --- |
| Pet species  (Canine) | 1.554 | 0.678 | 5.255 | 4.73 | 1.253-17.861 | 0.022* |
| Sharing of feeding area with other pets (Yes) | 1.703 | 0.862 | 3.908 | 5.49 | 1.02-29.74 | 0.048* |
| Presence of gastrointestinal associated symptoms (No) | 2.004 | 0.853 | 5.515 | 7.42 | 1.39-39.50 | 0.019* |

^a^ OR= odds ratio

*Significance level at *p*≤0.05

**STable 6.** ESBL genes identified in ESBL producing *E. coli* isolated from healthy pet cats and dogs

| ESBL genes | Isolates from dog | Isolates from cats |
| --- | --- | --- |
| *bla*_TEM_ | 11 | 2 |
| *bla*_CTX-M_ | 9 | 4 |
| *bla*_TEM/CTX-M_ | 4 | 2 |

**STable 7.** ESBL-genes detected in individual ESBL producing *E*s*cherichia coli* isolates from pet cats and dogs.

| Sample ID | Isolate | Bacterial Species | Host Species | ESBL Genes |
| --- | --- | --- | --- | --- |
| SC6 | 1  2  3 | *E. coli*  *E. coli*  *E. coli* | Cat | *bla*_TEM_  *bla*_TEM/CTX-M_  *bla*_TEM/CTX-M_ |
| NC7 | 2  3 | *E. coli*  *E. coli* | Cat | *bla*_CTX-M_  *bla*_CTX-M_ |
| CFC1 | 1  2  3 | *E. coli*  *E. coli*  *E. coli* | Cat | *bla*_CTX-M_  *bla*_CTX-M_  *bla*_CTX-M_ |
| CC10 | 1  2 | *E. coli*  *E. coli* | Cat | *bla*_CTX-M_  *bla*_TEM_ |
| PC3 | 1  2  3 | *E. coli*  *E. coli*  *E. coli* | Cat | *bla*_TEM/CTX-M_  *bla*_CTX-M_  *bla*_CTX-M_ |
| RC5 | 1  2  3 | *E. coli*  *E. coli*  *E. coli* | Cat | None of the observed genes detected in all three isolates |
| D5 | 1  2  3 | *E. coli*  *E. coli*  *E. coli* | Dog | *bla*_TEM_  *bla*_TEM/CTX-M_  *bla*_TEM/CTX-M_ |
| GD3 | 1  2 | *E. coli*  *E. coli* | Dog | *bla*_CTX-M_  *bla*_CTX-M_ |
| SD1 | 1  2  3 | *E. coli*  *E. coli*  *E. coli* | Dog | *bla*_CTX-M_  *bla*_CTX-M_  *bla*_CTX-M_ |
| GD5 | 1  2 | *E. coli*  *E. coli* | Dog | *bla*_TEM_  *bla*_TEM_ |
| GD7 | 1  2  3 | *E. coli*  *E. coli*  *E. coli* | Dog | *bla*_TEM/CTX-M_  *bla*_TEM_  *bla*_TEM_ |
| PJD1 | 2 | *E. coli* | Dog | *bla*_TEM_ |
| SSD2 | 1  2  3 | *E. coli*  *E. coli*  *E. coli* | Dog | *bla*_CTX-M_  *bla*_CTX-M_  *bla*_CTX-M_ |
| SSD5 | 2 | *E. coli* | Dog | *bla*_CTX-M_ |
| SSD8 | 3 | *E. coli* | Dog | *bla*_TEM/CTX-M_ |
| SSD11 | 1  3 | *E. coli*  *E. coli* | Dog | *bla*_TEM_  *bla*_TEM_ |
| SSD14 | 1  2  3 | *E. coli*  *E. coli*  *E. coli* | Dog | *bla*_TEM_  *bla*_TEM_  *bla*_TEM_ |

| **STable 1**: Multiple-drug resistance profile observed in ESBL producing *E. coli* isolated from cats and dogs in the Klang Valley. |
| --- |
| \| **Antibiotic resistance profile** \| **Proportion of resistance isolates (%)** \| \| **MDR ESBL-*E. coli* isolates**  **(≥3 antimicrobial category)** \| \| --- \| --- \| --- \| --- \| \| **Dog**  **(n=24)** \| **Cat**  **(n=16)** \|  \| \| **CL, AMP, TE, CN, CTX, ATM** \| 4 (16.6) \| 1 (6.2) \| Yes \| \| **CL, AMP, CTX** \| 0 (0) \| 4 (25.0) \| No \| \| **ENR, CL, AMP, TE, NA, CN, CTX, ATM** \| 2 (8.3) \| 1 (6.2) \| Yes \| \| **AMC, ENR, CL, AMP, TE, NA, CAZ, CTX, ATM** \| 3 (12.5) \| 0 (0) \| Yes \| \| **CL, AMP, TE, CTX, ATM** \| 0 (0) \| 2 (12.5) \| Yes \| \| **CL, AMP, TE, CTX** \| 0 (0) \| 2 (12.5) \| Yes \| \| **CL, AMP, TE, NA, CAZ, CTX, ATM** \| 0 (0) \| 1 (6.2) \| Yes \| \| **CL, AMP, TE, CAZ, CTX, ATM** \| 0 (0) \| 1 (6.2) \|  \| \| **CL, AMP, TE, CN, CAZ, CTX, ATM** \| 0 (0) \| 1 (12.5) \| Yes \| \| **AMC, ENR, CL, AMP, NA, CAZ, CTX, ATM** \| 2 (8.3) \| 0 (0) \| Yes \| \| **ENR, CL, AMP, TE, NA, CN, CTX** \| 1 (4.1) \| 0 (0) \| Yes \| \| **CL, AMP, ATM** \| 2 (8.3) \| 1(6.2) \| No \| \| **AMC, CL, AMP, TE, NA, CN, CAZ, CTX, ATM** \| 2 (8.3) \| 0 (0) \| Yes \| \| **CL, AMP, TE, CN, CTX** \| 1 (4.1) \| 1 (6.2) \| Yes \| \| **ENR, CL, AMP, TE, NA, CAZ, CTX, ATM** \| 1 (4.1) \| 0 (0) \| Yes \| \| **ENR, CL, AMP, TE, CN, CAZ, CTX, ATM** \| 1 (4.1) \| 0 (0) \| Yes \| \| **AMC, ENR, CL, AMP, TE, NA, CN, CAZ, CTZ, ATM** \| 1 (4.1) \| 0 (0) \| Yes \| \| **CL, AMP, TE, NA, CN, CTX, ATM** \| 1 (4.1) \| 0 (0) \| Yes \| \| **CL, AMP, TE, NA, CN, CAZ, CTX, ATM** \| 1 (4.1) \| 1 (6.2) \| Yes \| \| **AMC, CL, AMP, TE, CN, CTX** \| 1 (4.1) \| 0 (0) \| Yes \| \| **AMC, CL, AMP, TE, CN, CTX, ATM** \| 1 (4.1) \| 0 (0) \| Yes \|   AMC: Amoxicillin-clavulanic acid, ENR: Enrofloxacin, CL: Cephalexin, AMP: Ampicillin, TE: Tetracycline, NA: Nalidixic acid, CN: Gentamicin, CAZ: Ceftazidime, CTX: Cefotaxime and ATM: Aztreonam. |

| **STable 8.** Multiple-drug resistance profile observed in ESBL producing *E. coli* isolated from cats and dogs in the Klang Valley. |
| --- |
| \| **Antibiotic resistance profile** \| **Proportion of resistance isolates (%)** \| \| **MDR ESBL-*E. coli* isolates**  **(≥3 antimicrobial category)** \| \| --- \| --- \| --- \| --- \| \| **Dog**  **(n=24)** \| **Cat**  **(n=16)** \|  \| \| **CL, AMP, TE, CN, CTX, ATM** \| 4 (16.6) \| 1 (6.2) \| Yes \| \| **CL, AMP, CTX** \| 0 (0) \| 4 (25.0) \| No \| \| **ENR, CL, AMP, TE, NA, CN, CTX, ATM** \| 2 (8.3) \| 1 (6.2) \| Yes \| \| **AMC, ENR, CL, AMP, TE, NA, CAZ, CTX, ATM** \| 3 (12.5) \| 0 (0) \| Yes \| \| **CL, AMP, TE, CTX, ATM** \| 0 (0) \| 2 (12.5) \| Yes \| \| **CL, AMP, TE, CTX** \| 0 (0) \| 2 (12.5) \| Yes \| \| **CL, AMP, TE, NA, CAZ, CTX, ATM** \| 0 (0) \| 1 (6.2) \| Yes \| \| **CL, AMP, TE, CAZ, CTX, ATM** \| 0 (0) \| 1 (6.2) \|  \| \| **CL, AMP, TE, CN, CAZ, CTX, ATM** \| 0 (0) \| 1 (12.5) \| Yes \| \| **AMC, ENR, CL, AMP, NA, CAZ, CTX, ATM** \| 2 (8.3) \| 0 (0) \| Yes \| \| **ENR, CL, AMP, TE, NA, CN, CTX** \| 1 (4.1) \| 0 (0) \| Yes \| \| **CL, AMP, ATM** \| 2 (8.3) \| 1(6.2) \| No \| \| **AMC, CL, AMP, TE, NA, CN, CAZ, CTX, ATM** \| 2 (8.3) \| 0 (0) \| Yes \| \| **CL, AMP, TE, CN, CTX** \| 1 (4.1) \| 1 (6.2) \| Yes \| \| **ENR, CL, AMP, TE, NA, CAZ, CTX, ATM** \| 1 (4.1) \| 0 (0) \| Yes \| \| **ENR, CL, AMP, TE, CN, CAZ, CTX, ATM** \| 1 (4.1) \| 0 (0) \| Yes \| \| **AMC, ENR, CL, AMP, TE, NA, CN, CAZ, CTZ, ATM** \| 1 (4.1) \| 0 (0) \| Yes \| \| **CL, AMP, TE, NA, CN, CTX, ATM** \| 1 (4.1) \| 0 (0) \| Yes \| \| **CL, AMP, TE, NA, CN, CAZ, CTX, ATM** \| 1 (4.1) \| 1 (6.2) \| Yes \| \| **AMC, CL, AMP, TE, CN, CTX** \| 1 (4.1) \| 0 (0) \| Yes \| \| **AMC, CL, AMP, TE, CN, CTX, ATM** \| 1 (4.1) \| 0 (0) \| Yes \|   AMC: Amoxicillin-clavulanic acid, ENR: Enrofloxacin, CL: Cephalexin, AMP: Ampicillin, TE: Tetracycline, NA: Nalidixic acid, CN: Gentamicin, CAZ: Ceftazidime, CTX: Cefotaxime and ATM: Aztreonam. |
